# Evaluation of soybean sprouting growth vigor based on ZnONPs

**DOI:** 10.3389/fpls.2026.1746220

**Published:** 2026-03-16

**Authors:** Feng Pan, Qianyun Li, Silin Cao, Xiuqing Fu

**Affiliations:** 1 Institute of Smart Agriculture, Xinjiang Academy of Agricultural and Reclamation Science, Shihezi, China; 2 College of Engineering, Nanjing Agricultural University, Nanjing, China

**Keywords:** collection system, germination vigor assessment, soybean seed germination, YOLOv8-SEG improvement, ZnO nanoparticles

## Abstract

**Introduction:**

Nanoparticle-induced treatments can promote seed germination and improve germination potential under environmental stresses such as drought and salinity. This study aimed to investigate the effects of Zinc oxide nanoparticles (ZnONPs) on soybean seed germination and to develop a precise evaluation method.

**Methods:**

We developed a full-time sequence crop growth vitality monitoring system. Using germination rate and root length as primary evaluation indicators, we conducted full-time sequence germination vitality monitoring experiments on soybean seeds treated with ZnONPs. A dataset was constructed from images documenting embryonic root growth. The developed detection model was used to evaluate image detection accuracy during germination. Germination index and embryonic root length were also calculated. Further tests were performed on seeds exposed to 600 mg/L ZnONPs dispersion, followed by treatment with different concentrations of NaCl and PEG6000 solutions.

**Results:**

At a concentration of 600 mg/L ZnONPs dispersion, soybean seeds showed the highest germination rate (an increase of 28%) and the longest radicle length (an increase of 42%). Compared with deionized water, the 600 mg/L ZnONPs dispersion accelerated initial germination time, increased germination rate, and enhanced radicle length under low-concentration stress.

**Discussion:**

The results indicate that, at certain concentrations, ZnONPs dispersion positively influences soybean seed germination under varying salinity and drought conditions. We examined morphological and physiological changes in ZnONPs-treated seeds under stress, establishing a preliminary foundation for evaluating crop and variety vitality. These findings provide new insights that may contribute to improving soybean germination under simulated stress conditions, serving as a preliminary theoretical reference for potential applications in arid and saline environments.

## 1 Introduction

Soybean cultivation has a history spanning millennia and serves as a crucial source of protein and edible oil. In recent years, abiotic stresses such as salinity and drought have significantly reduced soybean productivity (Rhaman et al., 2022). Mineral nutrients, especially trace elements, play pivotal roles in plant growth and development. They act as cofactors for essential enzymes, contribute to biomolecular structures, and help plants withstand environmental stress. The seed germination stage, which is critical for crop establishment, is highly sensitive to the supply of certain minerals. Recent studies indicate that trace elements not only activate metabolism during germination but also enhance abiotic stress resistance via signaling pathways, thereby improving seed viability and seedling growth (Das and Adak, 2025). As Khot et al. (2012) suggested, nanoparticle-based approaches offer a novel strategy to mitigate adverse effects on soybean production, including during seed germination, seedling development, and abiotic stress. Zones have been reported as a potentially effective treatment for various environmental stresses due to their beneficial plant effects (Caldelas and Weiss, 2017). ZnONP treatment can improve plant morphology and physiological traits under both normal and stressed conditions (Mahmoud et al., 2019). Under salt and drought stress, ZnONP application increases enzyme activity, enhances nutrient uptake, and promotes photosynthesis in various plant species (Rizwan et al., 2019);. For example, ZnONP priming in *Vigna radiataL.* significantly improved growth parameters such as shoot length, root length, leaf number, and solute content (proline, total soluble sugars, and proteins). It also reduced malondialdehyde and hydrogen peroxide levels, strengthening antioxidant defenses (Mazhar et al., 2023). Overall, nanoparticle priming at certain concentrations can positively influence seed germination potential. Therefore, investigating the effects of nanoparticle priming on soybean seed germination under drought and salt stress, along with developing precise evaluation methods, is of considerable importance.

Seed vigor, one of the most representative indicators for assessing high−quality breeding, is defined as the comprehensive manifestation of seed characteristics that determine activity and performance during germination and seedling emergence (Carrera-Castaño et al., 2020). Quantifying the morphological changes in root growth induced by ZnONPs allows evaluation of seed germination potential. However, conventional methods for assessing germination vigor under nanoparticle induction often compromise seed integrity, require substantial manual effort, are time−consuming, and depend on operator subjective judgment. These factors limit the throughput, accuracy, reproducibility, and reliability of seed germination tests (Jahnke et al., 2016). Given these challenges, developing a high−precision, automated, and high−throughput method to evaluate soybean germination vigor is of significant research value. Advanced deep−learning algorithms can rapidly identify changes in soybean seed growth parameters, providing a more precise and intuitive assessment of seed germination vigor. Compared with traditional image processing and machine learning, instance segmentation enables fast and accurate identification and localization of objects within images. For example, the YOLOv8−peas model was used to determine germination rates of pea varieties and study their drought resistance (Jiang et al., 2023). Similarly, the YOLOv8−R model quantitatively assessed radish seed germination rates (Ouyang et al., 2024), while RootDetector, a neural network, detected bud and root length in micro−nodule images under field conditions (Peters et al., 2023). Additionally, MyROOT software combines bottom−up root tracking with hypocotyl detection to measure *Arabidopsis* root length (Betegón-Putze et al., 2019). To capture images throughout the entire crop growth cycle, we developed a full−time−sequence crop growth vigor monitoring system for high−throughput image acquisition during seed germination. Based on the YOLO architecture, the model was designed to detect and segment the seed radicle.

In summary, this study aimed to investigate the effects of nanoparticles on soybean seed germination vigor under drought and salt stress and to establish a precise evaluation method. To achieve this, we constructed a full-time-series dataset for monitoring soybean seed germination vigor using a non-destructive image acquisition system. A deep learning optimization model was developed to enable full-time-series tracking of key germination indicators, including germination rate, germination index, germination potential, and root length. Based on existing literature, we also discuss the mechanism by which ZnONPs affect seed germination, providing a theoretical framework for the observed phenotypic change. Germination tests were conducted on soybean seeds treated with ZnONPs dispersions at different concentrations. After comprehensive analysis, the optimal induction concentration was determined. The model was further validated through comparative performance tests in classifying, recognizing, and localizing soybean seed germination traits. Additionally, germination experiments were performed under varying drought and salinity conditions, with and without ZnONPs induction. The study analyzed changes in initial germination time, germination rate, and radicle length under different treatments, allowing precise evaluation of germination vitality across ZnONPs concentrations. This approach offers a novel strategy to evaluate and potentially enhance germination vigor of the tested soybean genotype under laboratory-induced laboratory-simulated drought and salinity stress.

## 2 Materials and methods

### 2.1 Seed germination phenotype collection system

**Figure 1:**
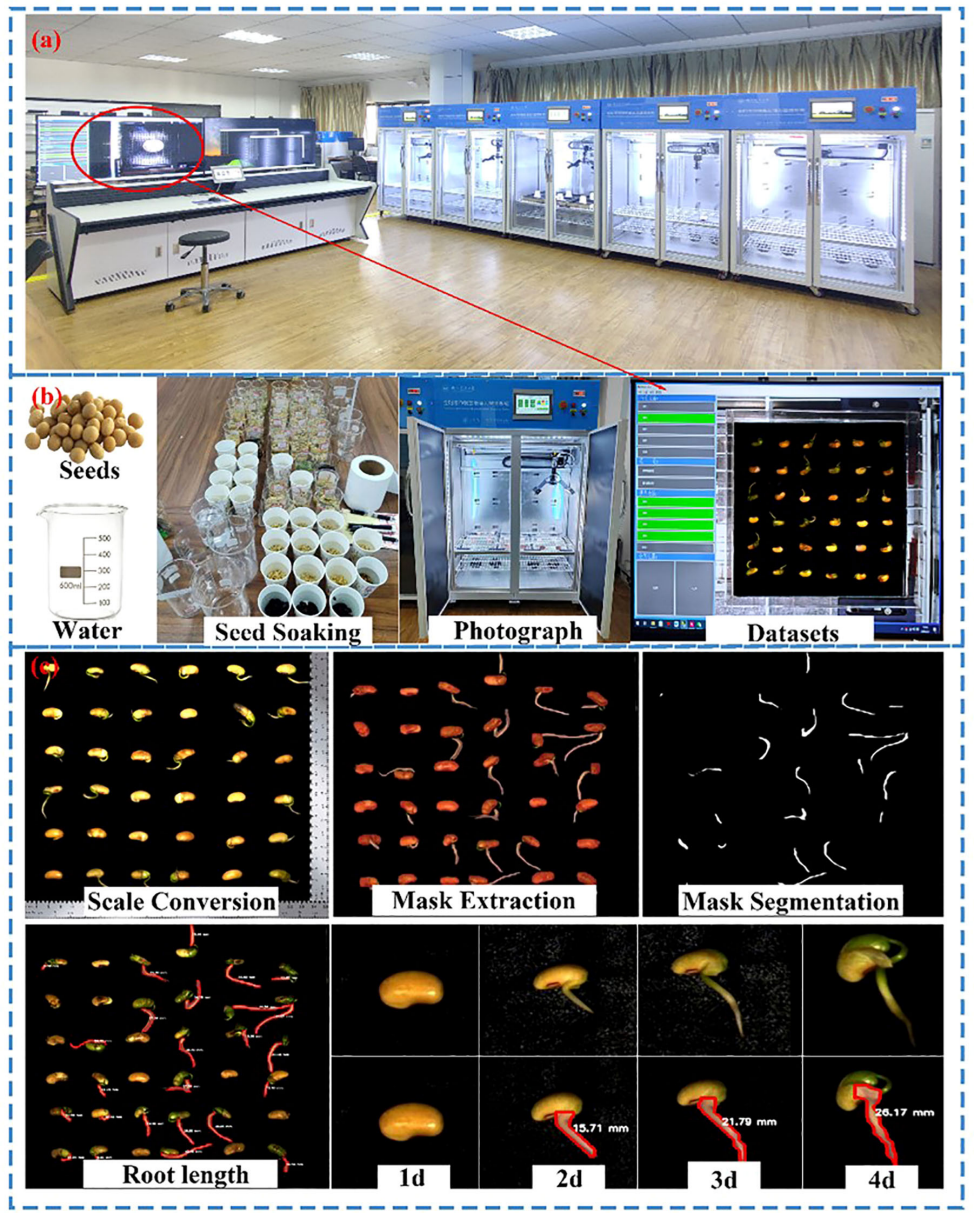
Physical diagram of the full-time sequence germination phenotype collection system. **(a)** Physical drawing of the incubator **(b)** Image acquisition **(c)** Root length calculation.

The full-time sequence crop growth vitality detection system constructed in the laboratory (**Figure 1**) comprises three modules: environmental control, image acquisition, and image storage. The environmental control module is based on a customized seed incubator. Its human-machine interface allows for real-time adjustment of temperature and humidity, as well as control of the LED light sources. The image acquisition module employs a rail system to control the camera’s horizontal movement along the X and Y axes above the chamber. Within each cycle, the camera moves sequentially to six predefined positions for stationary image capture. The camera is flanked by LED lights, which are activated during capture to ensure image clarity. The image storage module (**Figure 1c**) is designed to receive, store, and process the captured image, with the overall aim of building a dataset of soybean radicle phenotypes.

To develop an image dataset detection model for soybean seed germination, 216 plump and uniformly sized soybean Zhonghuang 13 seeds were selected. After preprocessing, two layers of pre−neutralized black filter paper were placed in each culture tray. The seeds were carefully arranged in six trays, with 36 seeds per tray. Seeds were then incubated at 25 ± 1 °C for a 96−hour full−time−series germination experiment under deionized water. The system systematically tracked seed growth dynamics from sowing to germination, documenting the entire developmental process. Using the Seed Germination Phenotyping Acquisition System (**Figure 1**), a total of 1,728 images were collected from the six trays to record the germination process. This dataset provided the foundation for subsequent target detection model development. To augment the dataset, reduce overfitting, enhance model robustness and generalization, and improve adaptability to varied conditions, image and label augmentation was performed through Gaussian blur, scaling, rotation, flipping, and brightness adjustment.

### 2.2 Training the germination image detection model

**Figure 2:**
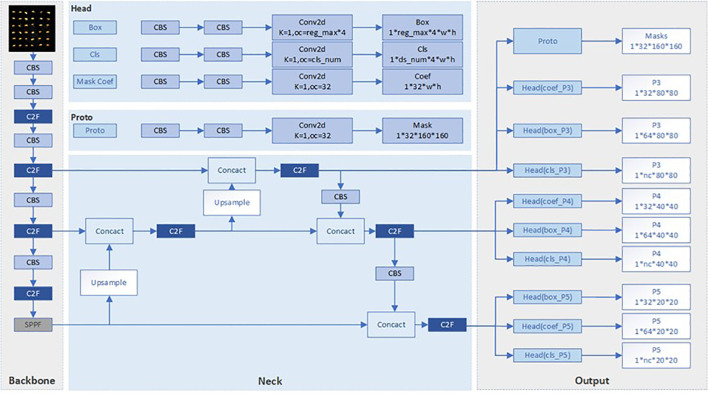
YOLOv8-segment model architecture diagram.

The objective of instance segmentation algorithms is to perform pixel-level segmentation of each distinct object in an image (Gu et al., 2022). Unlike semantic segmentation, instance segmentation requires not only identifying different semantic categories but also distinguishing individual instances within the same category. The YOLOv8-Seg model (**Figure 2**) is currently a mainstream method for seed germination detection, allowing precise instance segmentation analysis of crop roots and seed sections. As an advanced model in the field, YOLOv8-Seg achieves a balance between accuracy and speed by decomposing the instance segmentation task into two parallel subtasks.

Optimized models enhance the detection capability of minute germinating targets, achieving an effective balance between assessment accuracy and efficiency. This is crucial for advancing the technical level of seed germination vitality measurement.

#### 2.2.1 Evaluation metrics for YOLOv8 monitoring model

To provide a comprehensive evaluation of the soybean germination detection model’s performance, the mean average precision (mAPmask50) for instance segmentation and the mean average precision (mAPbox50) for object detection were selected in order to assess the model’s segmentation accuracy for masks and boxes, respectively. The mAP50 and mAP50-95 calculated using Equations 1 and 2. It is evident that higher average precision is indicative of enhanced model efficacy in the detection and segmentation of soybean radicles.

(1) $$mAP_{0.5}=\frac{1}{n_{c}}\int_{0}^{1}P(R)dR$$

(2) $${\text{mAP}}_{0.5-0.95}=\text{avg}({\text{mAP}}_{\text{i}}),\text{i}=0.5:0.05:0.95$$

#### 2.2.2 YOLOv8 monitoring model training parameter settings

This experiment utilized Anaconda3 to create a training virtual environment, employing Python 3.11, PyTorch 2.0.0, and Torchvision 0.15.1 as the code execution environment. CUDA and cuDNN were invoked to enhance training speed, the random seed was fixed at 0 during the model training process. The experimental environment and key hyperparameters are detailed in **Supplementary Table S1**.

#### 2.2.3 Optimization of the YOLOv8 monitoring model

To achieve an equilibrium between the precision of detection and the efficiency of runtime, three enhancements were implemented, drawing upon the principles of the YOLOv8-Seg-n instance segmentation model.

1. Replace conv with ghostconv: For high-throughput phenotyping tasks in agricultural seeds, Ghost Convolution (GhostConv) is introduced in network architecture design to optimize model efficiency. GhostConv is a cutting-edge lightweight convolution technique designed to reduce parameter redundancy and resource consumption. Its core principle involves extracting key information through finite intrinsic convolutions, then amplifying it into the required feature representations using inexpensive linear operators (such as depth wise separable convolutions). Compared to traditional convolutional layers, this structure significantly reduces computational complexity and parameter size (by approximately 30%-50%). It provides efficient computational support for the automated, precise identification and analysis of large-scale, multi-temporal seed development and germination images, substantially lowering computational and parameter demands for multi-image processing.

Assuming input channels are C, output channels are M, and kernel size isk×k:
Traditional convolution computational load:M×C×k×k
Ghost convolution computational load:
Intrinsic convolution:M^’×C×k×k (M^’=M/s, where s is the ghost feature multiplier)
Linear operation:M^’×(s-1)×d×d (d×d is the kernel size for the inexpensive operation, typically d=3) Total computational load is approximately 1/s of traditional convolution, with a similar reduction in parameter count.

**Figure 3:**
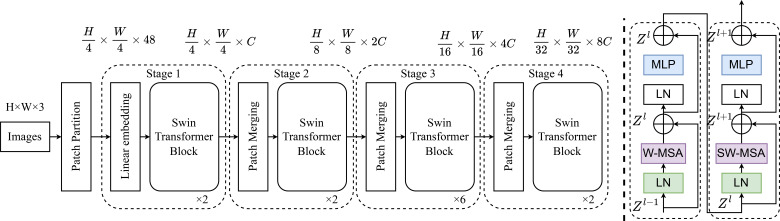
Overall structure of swin transformer.

**Figure 4:**
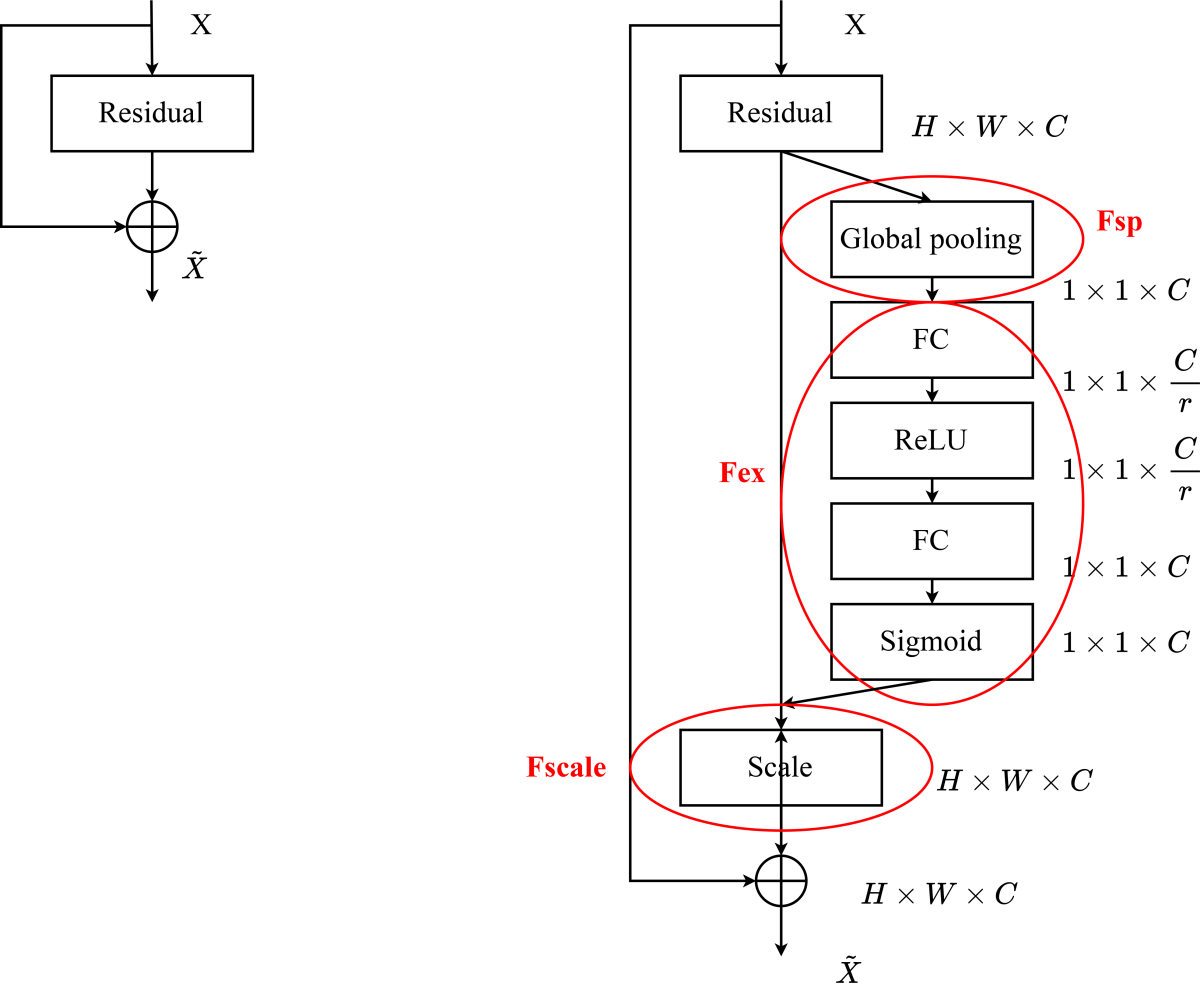
Structure of the SE attention module.

2. Integration of Swin Transformer Module: To achieve deeper modeling of complex spatial structures and multi-scale features in seed phenotypes, the Swin Transformer module (**Figure 3**) was further integrated. Drawing inspiration from hierarchical visual encoding, Swin Transformer efficiently aggregates local-to-global multi-level information through Patch Partition and Patch Merging. This architecture employs windowed multi-head self-attention mechanisms (W-MSA and SW-MSA), significantly enhancing the model’s ability to capture heterogeneous features such as seed micro-morphology, hypocotyl elongation, and abnormal germination. Additionally, the patch merging approach effectively avoids the loss of biologically critical information caused by traditional pooling, ensuring rigorous quantitative measurement of early seed developmental potential.

3. Adding the SE Attention Mechanism: To further enhance performance in detecting small seed targets, the Squeeze-and-Excitation (SE) attention mechanism was introduced into the model design (**Figure 4**). The SE attention module primarily consists of three core steps: Squeeze, Excitation, and Scale. The Squeeze operation performs global average pooling on the input feature map, compressing the response of each channel into a single global descriptive statistic. This effectively captures the global contextual information across all channels. Subsequently, the Excitation stage employs a two-layer fully connected neural network, supplemented by nonlinear activation functions (ReLU) and normalization functions (sigmoid), to model inter-channel dependencies and perform nonlinear recalibration. This ultimately generates normalized channel importance weight vectors. Finally, the Scale operation applies these weights adaptively to the original feature map through per-channel weighting, enhancing the expression of key features in critical channels while dynamically suppressing redundant or irrelevant information. In agricultural seed phenotyping applications, the SE module significantly enhances the model’s ability to characterize and distinguish fine-grained microstructures (individual seed morphology). This mechanism highlights highly relevant biological features, significantly improving detection accuracy for small target categories like seed germination and early development. It effectively supports high-throughput, automated seed quality evaluation and precision agricultural breeding research.

### 2.3 Root length calculation in YOLOv8 monitoring model

The instance segmentation model developed in this study can identify and segment the root of each soybean in complex backgrounds. However, the model’s output does not directly provide key biological parameters, such as root length. To address this, we applied contour detection-based image processing techniques to precisely define the segmentation boundaries of each soybean root. This allows root circumference to be calculated at the pixel level. Next, a scale calibration step converts pixel units into actual physical distances, enabling quantitative standardization across different experimental batches and imaging conditions. Finally, considering the natural curvature and non−linear growth of roots, half of the outer contour length of the extracted root mask is used as the estimated root length (L) for a single soybean plant (**Table 1**). This method is designed to minimize potential systematic underestimation errors that arise with traditional straight−line measurements.

**Table 1:** Root length measurement.

Parameter	k_1_	…	k_m_	…	k_n_
Pixel Points(Sp)	$k_{1}$	…	$\sum_{1}^{m}|k_{m},k_{m-1}|$	…	$\sum_{1}^{n}|k_{n},k_{n-1}|$
Perimeter(Sr)	$k_{1}/r$	…	$[\sum_{1}^{m}|k_{m},k_{m-1}|]/r$	…	$[\sum_{1}^{n}|k_{n},k_{n-1}|]/r$
Root Length(L)	$\frac{k_{1}}{r}/2$	…	$\frac{\left[\sum_{1}^{m}|k_{m},k_{m-1}|\right]}{r}/2$	…	$\frac{\left[\sum_{1}^{n}|k_{n},k_{n-1}|\right]}{r}/2$

To evaluate the accuracy of the root length estimation method, we compared it with manual measurements. A total of 100 embryonic root samples were randomly selected from images taken at different time points across multiple experimental groups, including the control and various stress treatments. It is important to note that these samples were selected from the early germination phase (0–96 h), where the root morphology is characterized primarily by the elongation of the radicle with limited lateral branching, ensuring the suitability of the contour-based estimation method. Two experienced researchers manually traced the curved paths of these roots using ImageJ software to obtain reference root length values. The same 100 root images were then processed with our YOLOv8-SEGCAL model to automatically generate segmentation masks and compute estimated root lengths. The accuracy of the method was quantified by calculating the linear regression coefficient of determination (R²) between the manually measured and automatically estimated root length data.

### 2.4 Experimental mechanism of ZnONPs’ influence on seed germination

**Figure 5:**
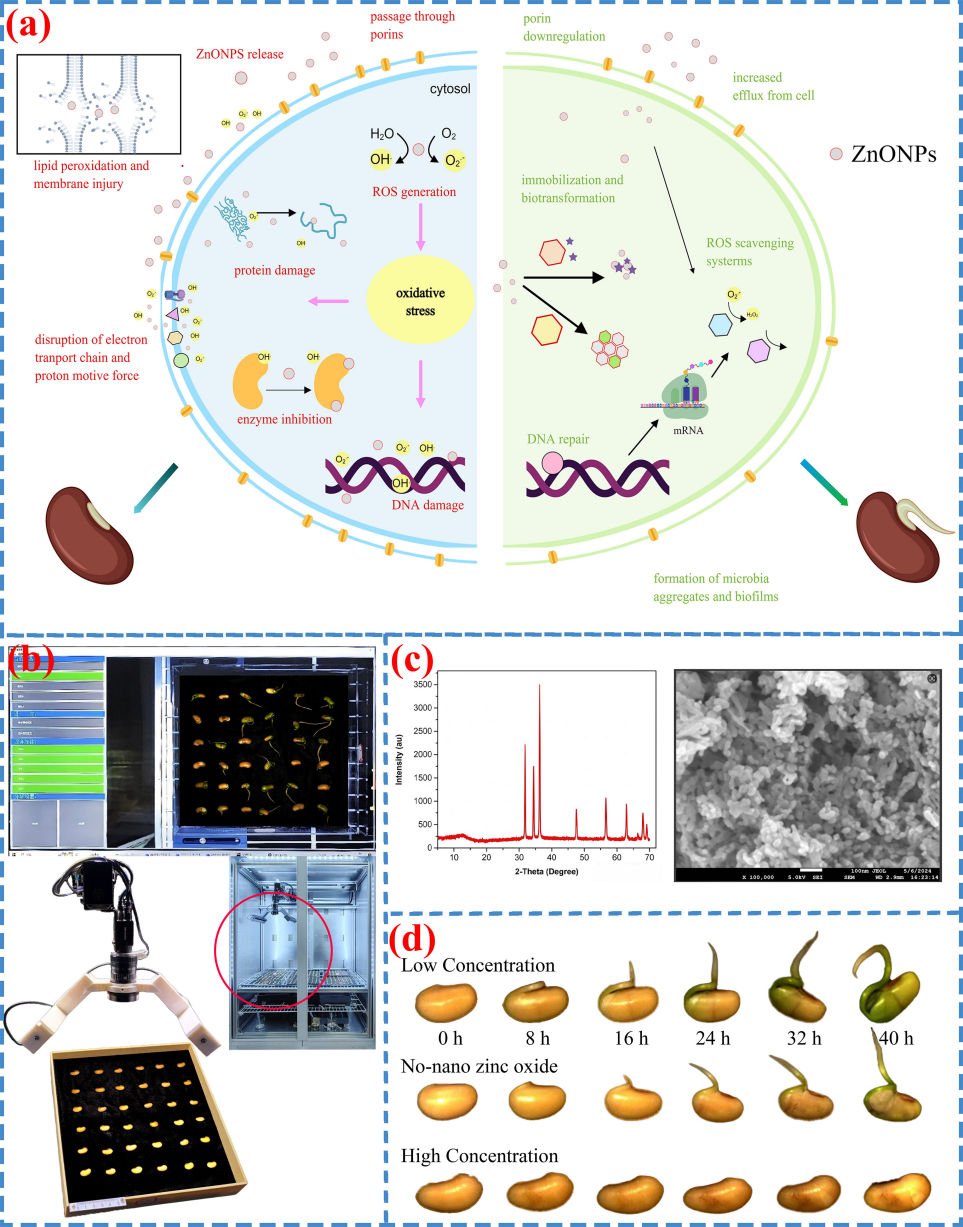
Results at different ZnONPs concentrations. **(a)** Mechanism of ZnONPs influence **(b)** Image acquisition **(c)** ZnONPs characterization **(d)** Treatment.

Existing research and phenotypic associations suggest the hypothesis that nano−zinc oxide particles can release zinc ions, which potentially play a role in cell division and proliferation by participating in the synthesis and accumulation of auxin and indole−3−acetic acid (**Figure 5**). Under stress conditions, zinc binds to sulfhydryl groups and phospholipids, helping to maintain membrane stability and integrity in soybean. Zinc application also enhances the uptake of both micronutrients and macronutrients under stress. To minimize experimental errors and ensure reagent quality, we characterized the synthesized ZnONPs. X−ray diffraction (XRD) was used to evaluate crystal structure and phase purity (Stefanos et al., 2018). The resulting pattern (**Figure 5c**) showed no impurity peaks, confirming high phase purity. Scanning electron microscopy (SEM) and transmission electron microscopy (TEM) were employed to analyze surface morphology and size distribution. SEM images revealed the nanoscale morphology (Das et al., 2025), while TEM further confirmed the spherical shape of the ZnONPs, with a size range of 15–20 nm.

Nano−zinc oxide treatment can improve plant morphology and physiological traits under both normal and stress conditions (Mahmoud et al., 2020). This study aimed to investigate how different concentrations of ZnONPs dispersions affect soybean seed germination vigor. Germination tests were performed on soybean seeds exposed to varying ZnONPs concentrations (**Table 2**). Each treatment and control group contained 36 seeds per petri dish, with three replicates per concentration to ensure result reliability. After seed pretreatment, petri dishes were placed in the collection system at 25 ± 1 °C to promote germination. Throughout the experiment, 10 mL of deionized water or the corresponding treatment solution was added daily to each dish to maintain consistent conditions. In total, 648 seeds were used, yielding 1,728 images recorded over 96 h to document the germination process.

**Table 2:** Experimental parameters of soybean seed germination under ZnONPs dispersion stress.

Parameters	Test
Solution	ZnO NPs
Concentration	0 200mg/L 400mg/L 600mg/L 800mg/L 1000mg/L
Number of seeds per plot	36
Number of repeated trials	3
Soaking time	96h
Incubation time	96h
Incubation temperature	25 ± 1°C
Time between shots	15min
Image resolution	5472×3648
Image format	.jpg

### 2.5 Application

Salt and drought are two major environmental stresses that severely limit plant growth and crop yield (Masmoudi et al., 2021). Their effects on crop growth vary with stress intensity, particularly in biomass accumulation patterns. Establishing quantitative relationships between salinity levels and crop growth is crucial for evaluating crop and varietal vigor, as well as for selecting appropriate assessment protocols potentially suited to different soil conditions, subject to further multi-genotype testing and field validation. Based on previous experiments showing that 600 mg/L ZnONPs dispersion best promoted soybean seed development, this concentration was selected for further testing under salt and drought stress. Experimental groups included 600 mg/L ZnONPs dispersion combined with different concentrations of NaCl or PEG6000 solutions, which were compared with corresponding stress treatments without ZnONPs. To quantify soybean growth responses, germination tests were performed on four seed groups (**Table 3**). Each treatment and control group contained 36 seeds per petri dish, with three replicates per concentration to ensure reliability. After pretreatment, dishes were placed in the collection system at 25 ± 1 °C. Throughout the experiment, 10 mL of deionized water or the corresponding treatment solution was added daily to maintain consistent conditions. A total of 6,912 images were captured over 96 h to document germination.

**Table 3:** Control experiment parameters for seed germination under multiple stress treatments.

Parameters	Test1	Test2	Test3	Test4
Solution	NaCl	NaCl+600mg/LZnONPs	PEG6000	PEG6000 + 600mg/LZnONPs
Concentration	0 mmol/L 40 mmol/L 80 mmol/L 120 mmol/L 160 mmol/L 200 mmol/L	0 mmol/L NaCl+600mg/LZnONPs 40 mmol/L NaCl+600mg/LZnONPs 80 mmol/L NaCl+600mg/LZnONPs 120 mmol/L NaCl+600mg/LZnONPs 160 mmol/L NaCl+600mg/LZnONPs 200 mmol/L NaCl+600mg/LZnONPs	0% 2% 4% 6% 8% 10%	0% PEG6000 + 600mg/LZnONPs 2% PEG6000 + 600mg/LZnONPs 4% PEG6000 + 600mg/LZnONPs 6% PEG6000 + 600mg/LZnONPs 8% PEG6000 + 600mg/LZnONPs 10% PEG6000 + 600mg/LZnONPs
Number of seeds per plot	36	36	36	36
Number of repeated trials	3	3	3	3
Soaking time	10	10	10	10
Incubation time	96h	96h	96h	96h
Incubation temperature	25 ± 1°C	25 ± 1°C	25 ± 1°C	25 ± 1°C
Time between shots	20min	20min	20min	20min
Image resolution	5472×3648	5472×3648	5472×3648	5472×3648
Image format	.jpg	.jpg	.jpg	.jpg

## 3 Results

### 3.1 Model optimization

To demonstrate the superiority of the proposed model in detecting and segmenting soybean radicles when compared with other models, comparative experiments were conducted with YOLOv5, YOLOv7, YOLOv8, and the proposed model (**Table 4**). The results were trained under identical configuration environments and hyperparameter settings. The model under consideration achieved the highest detection mean average precision (mAP) and segmentation mask mAP. The mAP50 metric achieved a detection rate of 99.4%, while the mAP50–95 metric attained 72.9%. For the segmentation masks, mAP50 attained 88.5%, and mAP50–95 reached 37%, thus demonstrating superiority over all other models. In comparison with the unmodified YOLOv8, the detection mAP (50-95) demonstrated an enhancement of approximately 2%, whilst the segmentation mask mAP (50-95) exhibited a 4% increase. YOLOv5 offers the most lightweight model, but its accuracy for soybean radicle segmentation is poor, achieving only 61.3% mAP50 and 15.1% mAP50–95 for segmentation. YOLOv8SEGCAL attains merely one-fifth of the GFLOPS achieved by YOLOv7. However, its detection mAP50 exhibits a 3.7% increase, while its segmentation mask mAP (50-95) demonstrates a 21.9% enhancement in comparison to YOLOv7.

**Table 4:** Performance comparison of YOLO series models.

Models	mAP^box50^	mAP^box50-95^	mAP^mask50^	mAP^mask50-95^
YOLOv5	95.7	52.6	61.3	15.1
YOLOv7	97.4	62.5	84.7	27.1
YOLOv8	97.6	70.7	85.0	35.9
YOLOv8SEGCAL	**99.4**	**72.9**	**88.5**	**37.0**

Bold numbers indicate the optimized model parameters.

### 3.2 Verification of root length calculation method

**Figure 6:**
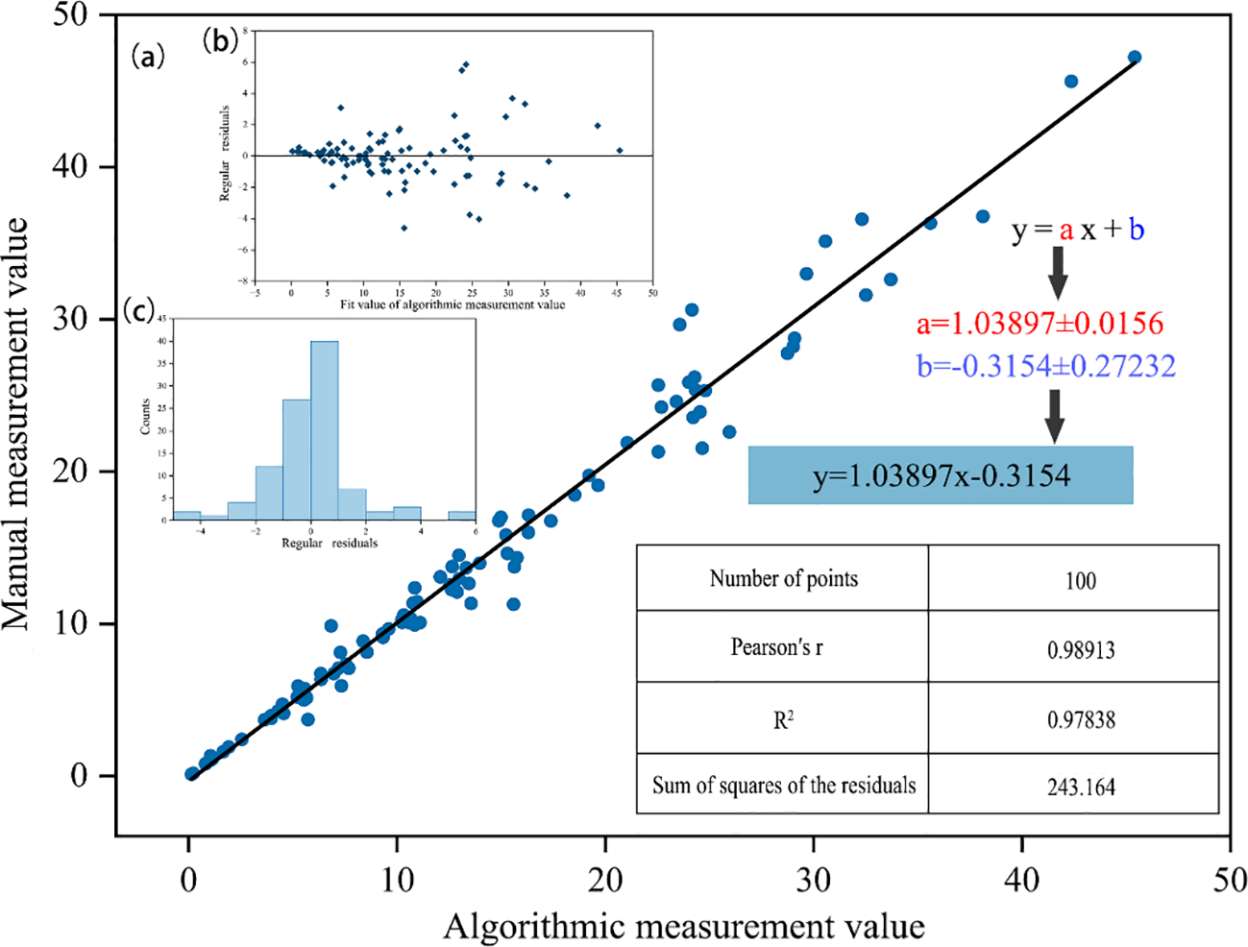
Correlation analysis between the detected values of YOLOv8SEGCAL algorithm and the manually measured values. **(a)** Fitted line chart **(b)** Residual scatter plot **(c)** Normal distribution chart.

Root length estimates from this study were compared with manual measurements (**Figure 6**) to assess data consistency. Linear regression analysis showed an R²value of 0.978, indicating high agreement between estimated and measured root lengths. The slope was close to 1, demonstrating that the method is accurate and reliably captures relative trends in embryonic root length. This validation confirms the model’s reliability specifically for early-stage germination assessments where root entanglement and complex branching are minimal **Figure 6**.

### 3.3 ZnONPs dispersion solution induces

**Figure 7:**
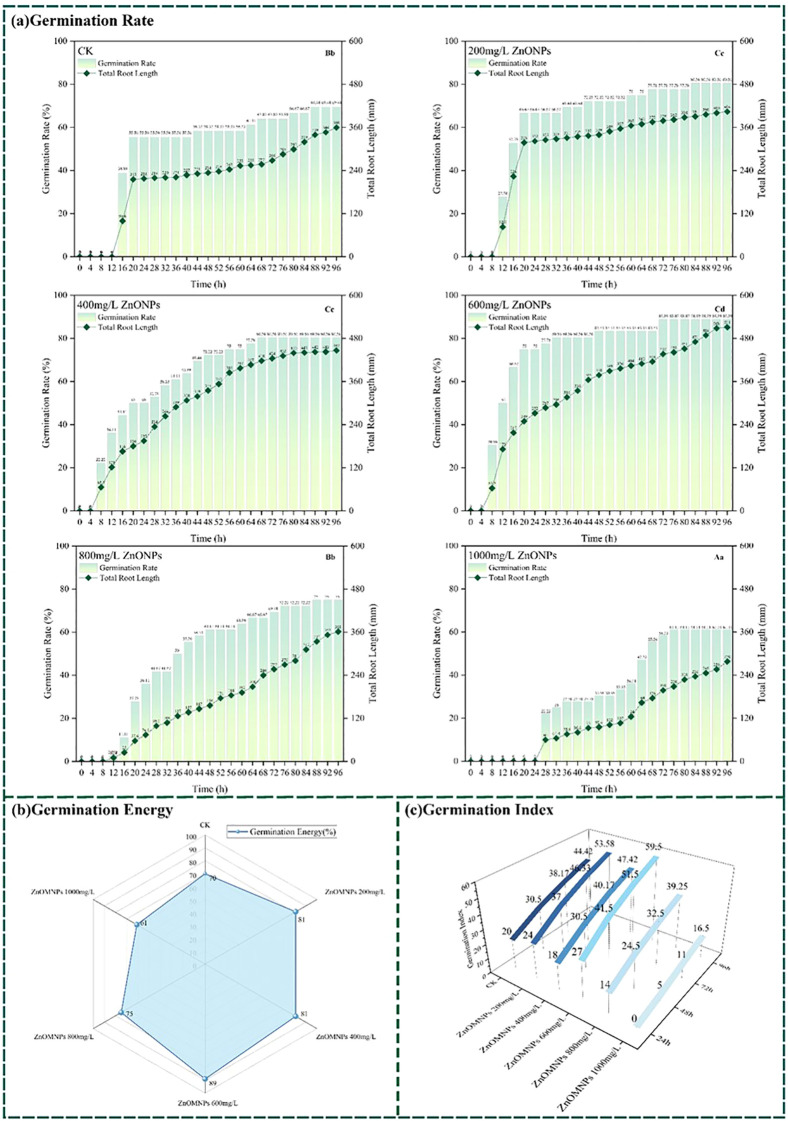
Changes in germination rate, germination vigor, and germination index of soybean seeds under different ZnONPs dispersion conditions **(a)** Germination rate and total root length of seeds under different ZnONPs concentrations. **(b)** Germination energy of seeds under different treatments. **(c)** Germination index of seeds under different treatments [36 seeds/group, biological replicates n=3. Values are expressed as means. Uppercase letters indicate comparisons among germination rates, and lowercase letters indicate comparisons among root lengths, both based on Tukey’s test (p<0.05)].

A full-time-series germination vigor monitoring experiment was conducted on soybean seeds induced by ZnONPs dispersions. A total of 1,728 images documenting the entire germination process were analyzed using the YOLOv8SEGCAL model. The developed YOLOv8SEGCAL detection model was employed to evaluate the germination rate and root length as primary indicators. The data presented in **Figure 7** quantifies the germination rate, germination vigor, and germination index of soybean seeds under the control group (CK) and different ZnONPs dispersion concentrations (**Figure 7**).

Prior to ANOVA, the assumptions of normality and homogeneity of variance were assessed using the Shapiro-Wilk test and Levene’s test, respectively. Although some experimental groups exhibited deviations from normal distribution or homogeneity of variance (p < 0.05), one-way ANOVA was performed followed by Tukey’s HSD *post-hoc* test. Despite some deviations from strict normality or homogeneity of variance in a few groups, we proceeded with ANOVA followed by Tukey’s HSD test, relying on the robustness of the F-statistic given our balanced experimental design (equal sample sizes across all treatments). (1) ANOVA revealed highly significant differences in germination rates among treatments (p < 0.001). Germination rate at 1000 mg/L was significantly lower than all other treatment (p < 0.05), representing the lowest value. The 800 mg/L treatment did not differ significantly from the 0 mg/L control (p > 0.05). but both were significantly lower than the 200, 400, and 600 mg/L treatments. Germination rates at 200, 400, and 600 mg/L did not differ significantly from each other (p > 0.05). yet all were significantly higher than the 0, 800, and 1000 mg/L treatments. The highest germination rate occurred at 600 mg/L. (2) Similarly, treatments had a highly significant effect on total root length (p < 0.001). The 1000 mg/L group showed the shortest mean root length, significantly lower than all others (p < 0.05). The 800 mg/L group did not differ from the 0 mg/L control, but both were significantly shorter than the 200, 400, and 600 mg/L groups. Root lengths at 200 and 400 mg/L were statistically similar, both longer than the 0, 800, and 1000 mg/L groups but shorter than the 600 mg/L treatment. The longest root length was observed at 600 mg/L ZnONPs.

**Figure 8:**
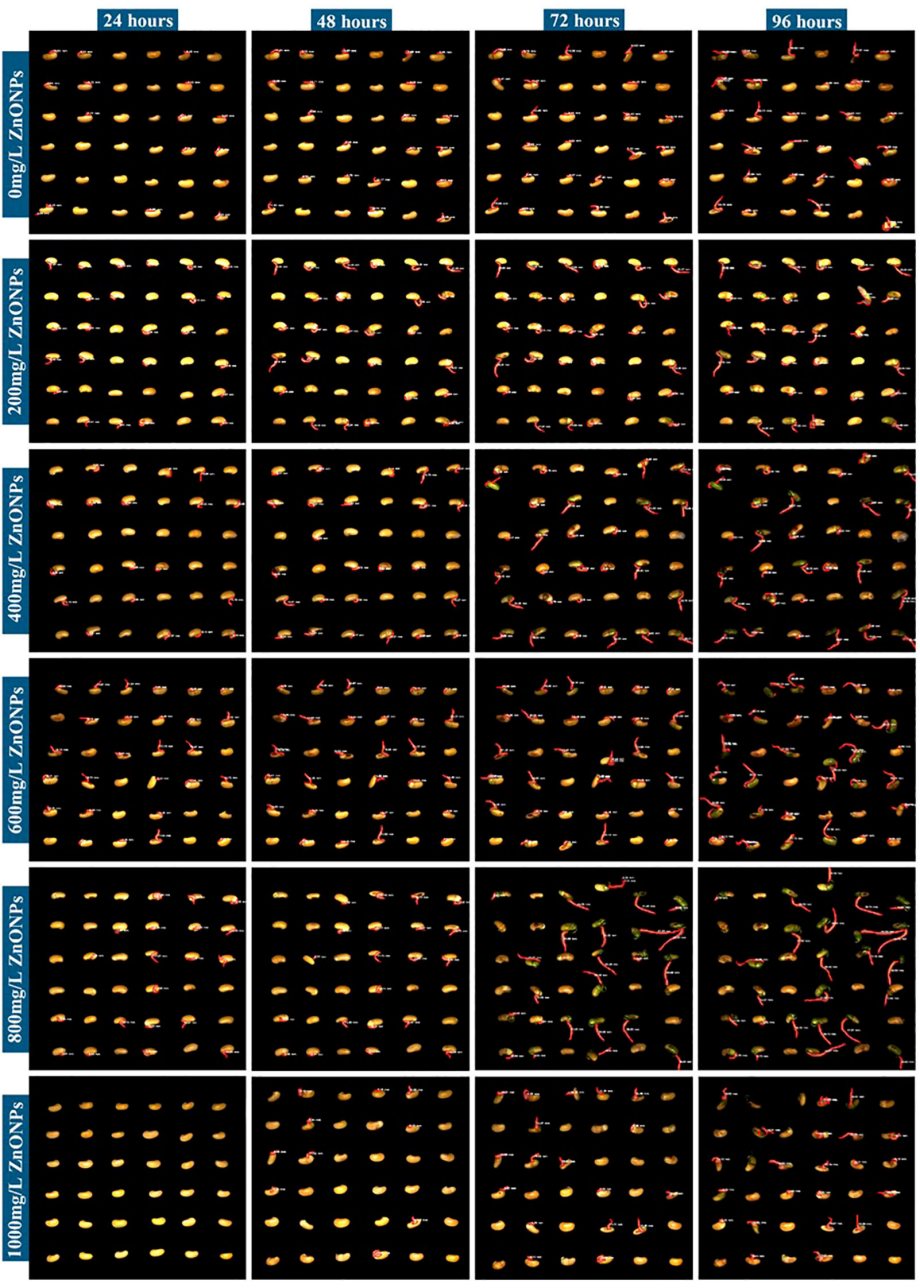
Germination process images of soybean seeds at different ZnONPs concentrations over 96 hours(36 seeds/group).

The experiments revealed that different concentrations of ZnONPs exerted varying effects on crop growth. It is noteworthy that the 600 mg/L concentration significantly promoted soybean seed development. In comparison with the CK, soybean seeds that had been subjected to a dispersion of 600 mg/L ZnONPs demonstrated earlier germination onset, higher germination rates, and increased total root length. The following images depict the germination process of soybean seeds that were treated with a dispersion of ZnONPs at a concentration of 600 mg/L. The processed images are shown below (**Figure 8**).

### 3.4 Soybean seed germination vitality test

**Figure 9:**
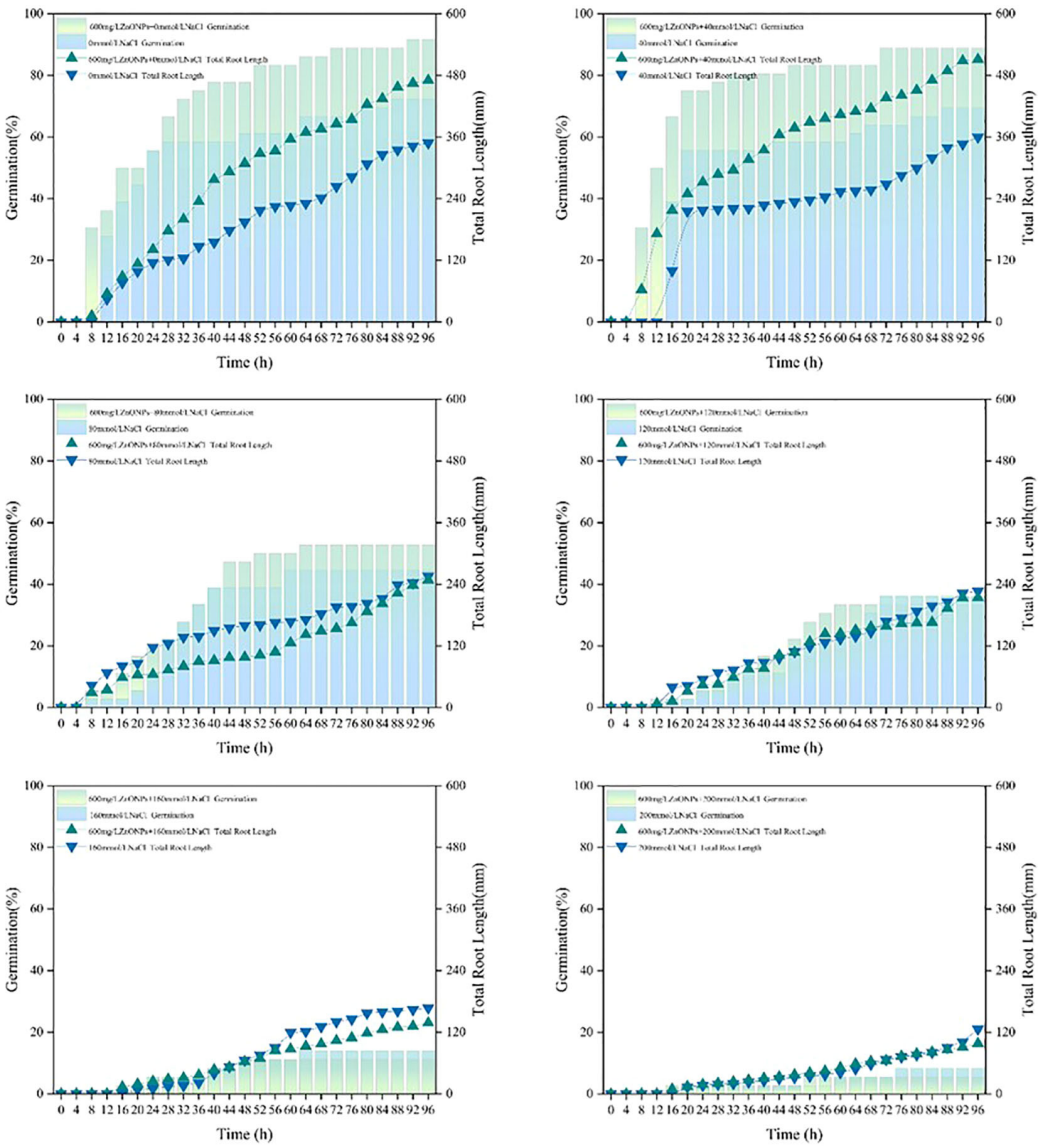
Effects of combined treatments with different concentrations of ZnONPs and NaCl on seed germination rate (column) and total root length (line) [36 seeds/group, biological replicates n=3. Data are expressed as means. Two-way ANOVA was used to examine the significant effects of ZnONPs concentration, NaCl concentration, and their interaction on germination rate and root length. Tukey’s HSD *post-hoc* test was performed to analyze significant differences among different treatment groups (p < 0.05)].

Using the YOLOv8SEGCAL model, we analyzed 6,912 images of soybean germination across 24 experimental groups. The results indicated that a 600 mg/L ZnONPs dispersion produced germination rates and root lengths comparable to those in control groups treated with equivalent concentrations of NaCl or PEG6000 solutions (**Figure 9**).

Prior to ANOVA, the assumptions of normality and homogeneity of variance were assessed using the Shapiro-Wilk test and Levene’s test, respectively. Although some experimental groups exhibited deviations from normal distribution or homogeneity of variance (p < 0.05), two-way ANOVA was performed followed by Tukey’s HSD *post-hoc* test. Despite some deviations from strict normality or homogeneity of variance in a few groups, we proceeded with ANOVA followed by Tukey’s HSD test, relying on the robustness of the F-statistic given our balanced experimental design (equal sample sizes across all treatments). (1) NaCl concentration, together with ZnONPs, significantly affected the final germination rate of soybean seeds (p < 0.01). The main effect of NaCl concentration was most pronounced, while the effect of ZnONPs depended on NaCl levels. Overall germination rate decreased as NaCl concentration increased. At 0 mmol/L NaCl, seeds treated with ZnONPs showed a higher mean germination rate than the control; this difference increased under mild salt stress (40 mmol/L). At higher salt concentrations (≥120 mmol/L), germination rates did not differ significantly between ZnONPs−treated and untreated seeds. Without considering interactions, ZnONPs−treated groups had a significantly higher average germination rate than untreated groups (p < 0.001). Germination rates decreased significantly with rising NaCl concentration, with significant differences observed among all salt levels except between 0 and 40 mmol/L (p < 0.05). The interaction between NaCl concentration and ZnONPs treatment also significantly influenced root length (p < 0.01). NaCl concentration was the primary factor affecting root length, which declined markedly from 0 to 200 mmol/L. Root lengths at 0 and 40 mmol/L did not differ significantly but were both longer than those at ≥80 mmol/L. ZnONPs treatment also had a significant main effect: mean root length was greater with 600 mg/L ZnONPs than without. Notably, a significant interaction indicated that ZnONPs increased root length under no−salt (0 mmol/L) and mild−salt (40 mmol/L) conditions, but not at salt concentrations of 80 mmol/L or higher.

**Figure 10:**
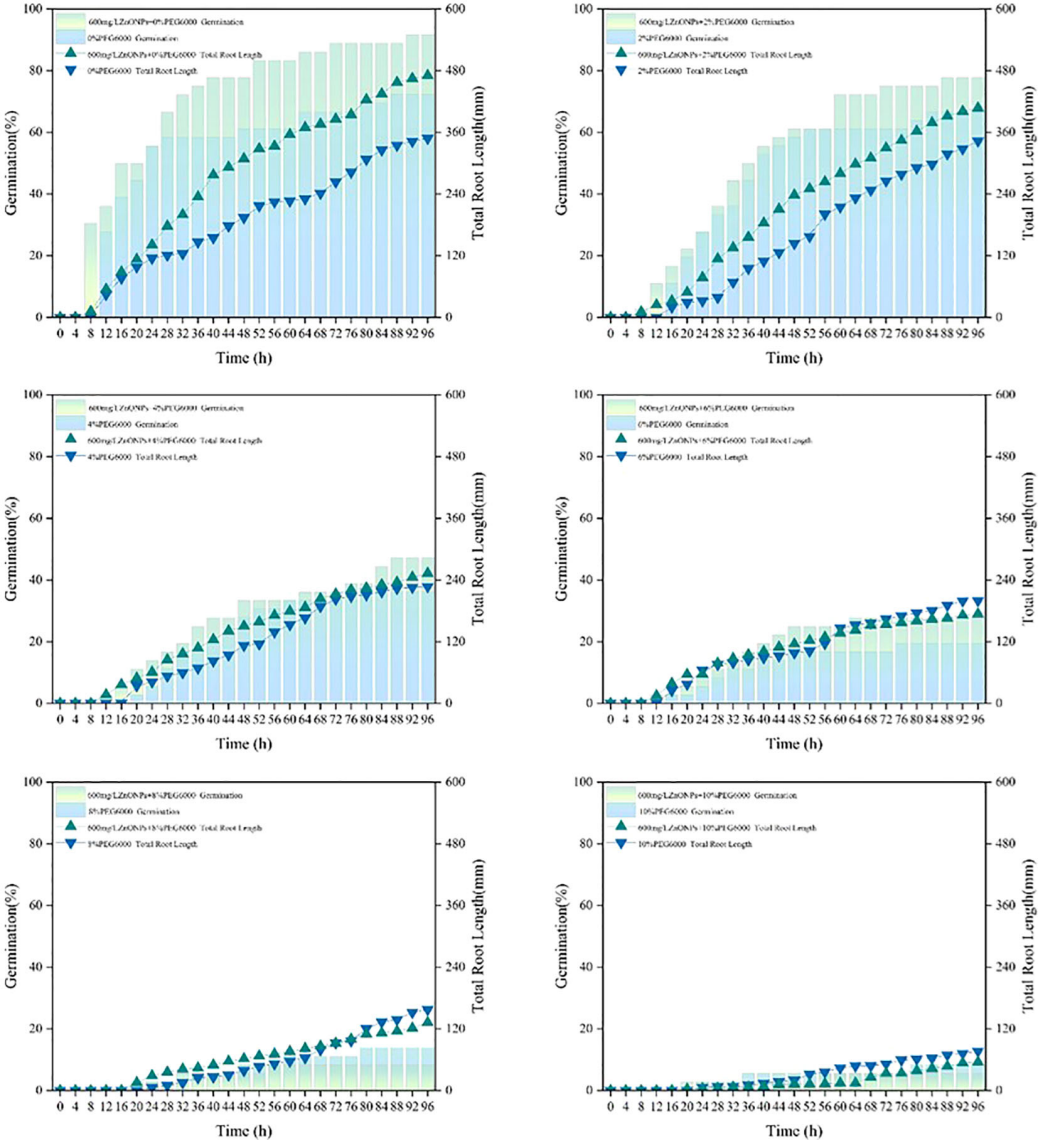
Effects of combined treatments with different concentrations of ZnONPs and PEG6000 on seed germination rate (column) and total root length (line) [36 seeds/group, biological replicates n=3. Data are expressed as means. Two-way ANOVA was used to examine the significant effects of ZnONPs concentration, PEG6000 concentration, and their interaction on germination rate and root length. Tukey’s HSD *post-hoc* test was performed to analyze significant differences among different treatment groups (p < 0.05)].

The experimental findings suggest that soybean seeds treated with a dispersion of 600 mg/L ZnONPs exhibited earlier germination time, higher germination rate, and increased total root length in ion-free water (CK) at concentrations of 40 mmol/L, 80 mmol/L, 120 mmol/L, 160 mmol/L, and 200 mmol/L of NaCl solutions. Conversely, high concentrations of NaCl resulted in a reduction in both the germination rate and total root length. The observed trend is consistent with the documented changes in germination rate and root length (**Figure 10**).

Prior to ANOVA, the assumptions of normality and homogeneity of variance were assessed using the Shapiro-Wilk test and Levene’s test, respectively. Although some experimental groups exhibited deviations from normal distribution or homogeneity of variance (p < 0.05), two-way ANOVA was performed followed by Tukey’s HSD *post-hoc* test. Despite some deviations from strict normality or homogeneity of variance in a few groups, we proceeded with ANOVA followed by Tukey’s HSD test, relying on the robustness of the F-statistic given our balanced experimental design (equal sample sizes across all treatments). (1)A significant interaction was observed between ZnONPs treatment and PEG6000 concentration. PEG6000 concentration had a highly significant main effect on germination rate (p < 0.001), indicating it was the predominant factor. ZnONPs treatment also significantly influenced germination rate (p < 0.01), with its effect depending on PEG6000 levels. Overall, germination rate decreased significantly as PEG6000 concentration increased.

Under no stress (0% PEG6000) and mild stress (2% PEG6000), ZnONPs addition significantly improved germination. Under moderate to severe stress (4–8% PEG6000), mean germination rates in ZnONPs−treated groups showed slight but non−significant increases compared with the control. (2)A strong interaction was also found between ZnONPs treatment and PEG6000 concentration for root length. PEG6000 concentration exerted a highly significant main effect on root length (p < 0.001), and ZnONPs treatment showed a significant main effect (p < 0.01). Root length decreased significantly with rising PEG6000 concentration. In general, mean root length was notably greater in the 600 mg/L ZnONPs−treated group than in the untreated group, suggesting that ZnONPs promoted root growth under low−concentration stress within the tested range.

**Figure 11:**
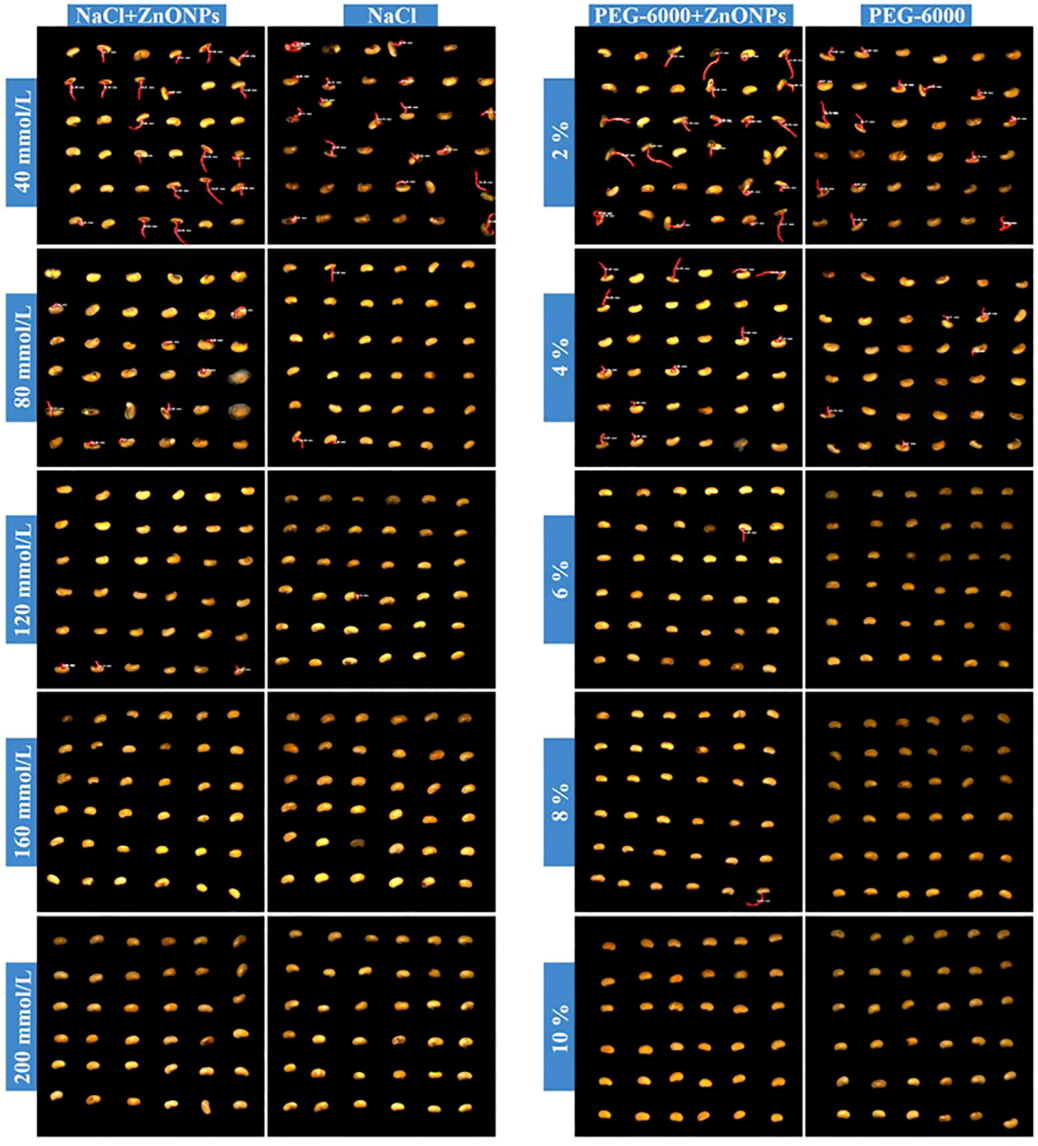
Germination process images of soybean seeds under different ZnONPs conditions subjected to NaCl and PEG6000 stress at varying concentrations(36 seeds/group).

The experimental findings suggest that soybean seeds which had been treated with a dispersion of ZnONPs at concentrations of 600 mg/L in ion-free water (CK), as well as in 2%, 4%, 6%, 8% and 10% PEG6000 solutions, exhibited earlier germination, higher germination rates, and increased total root length at low concentrations of PEG6000. Conversely, high concentrations resulted in decreased germination rates and reduced total root length. As demonstrated in **Figure 8**, the observed trends are in alignment with the changes in germination rate and root length. The following images illustrate the germination process of soybean seeds in the presence and absence of 600 mg/L ZnONPs dispersion, subjected to varying concentrations of NaCl and PEG6000 (**Figure 11**).

## 4 Conclusions

Soybean germplasm resources and breeding technologies have gained global attention due to the crop’s importance as a major food source. This study developed a high−throughput monitoring system incorporating an enhanced YOLOv8 model to precisely evaluate the germination−promoting effects of ZnONPs on soybean seeds. Results showed that under normal and low−stress conditions, 600 mg/L ZnONPs dispersion significantly improved germination rates, accelerated germination, and increased radicle length. This enhancement is hypothesized to be associated with the sustained release of Zn^2+^ ions, which could potentially serve as cofactors for key enzymes theoretically involved in seed reserve mobilization and antioxidant defense. The observed phenotypic mitigation of salt-drought stress suppression leads to the speculation that ZnONPs pretreatment might theoretically contribute to membrane stability, possibly through mechanisms such as the potential regulation of key enzyme activities (e.g., superoxide dismutase) and the reduction of oxidative damage.

Nevertheless, this study has several limitations. First, conclusions are based on laboratory−controlled conditions using a single soybean variety (Zhonghuang 13). The optimal concentration (600 mg/L) requires validation for other varieties and under field conditions influenced by soil and climate factors. Second, the study focused on phenotypic evaluation methods for germination vigor and did not include direct physiological or biochemical measurements (e.g., antioxidant activity, reactive oxygen species levels, or zinc uptake). Therefore, the discussion of ZnONPs ‘mechanism relies on existing literature and remains hypothetical. Future studies should incorporate biochemical experiments to verify the proposed mechanisms. Third, the improved algorithm may encounter recognition errors when identifying complex scenarios involving root entanglement during the late budding stage, necessitating further optimization in the future to accommodate a wider range of scenarios. Additionally, while positive effects during germination were documented, the study did not assess ZnONPs accumulation in soil ecosystems or their potential long−term environmental risks to plant growth and soil microbial communities—a key aspect related to initial environmental risk assessment objectives. Finally, regarding statistical analysis, preliminary tests indicated that certain data subsets deviated from the strict assumptions of normality and homogeneity of variance required for ANOVA. While the balanced design mitigates this issue, these statistical deviations represent a limitation that suggests future studies could employ non-parametric methods or larger sample sizes to further validate the significance of the findings.

Future research should: (1) validate ZnONPs pretreatment effects across different soybean genotypes and in field trials; (2) supplement measurements of reactive oxygen species, antioxidant enzyme activity, and zinc ion uptake to clarify physiological and biochemical mechanisms; (3) investigate the long−term fate of ZnONPs in agricultural soils and their impact on soil health, as well as potential synergies with other sustainable agronomic practices for abiotic stress resilience. (4) improve the algorithm to enable recognition in more scenarios.

Despite these limitations, this study proposes a novel and efficient strategy for rapidly assessing and enhancing seed viability. The integrated phenomics−deep learning platform offers a valuable tool for screening crop varieties with improved germination−stage stress tolerance. The optimized ZnONPs pretreatment demonstrated efficacy in a laboratory setting for supporting the germination of the Zhonghuang 13 variety under stress. These results suggest it has the potential to serve as a seed treatment technology; however, further validation across diverse genotypes and field trials is required to confirm its applicability in actual arid and saline-alkali regions, thereby contributing to sustainable soybean production under challenging climatic conditions.

## Data Availability

The raw data supporting the conclusions of this article will be made available by the authors, without undue reservation.

## Supplementary material

Supplementary Table 1 Training environment and main hyperparameters of the sprouting image detection model.
