# Supplementary material for: Evaluation of soybean sprouting growth vigor based on ZnONPs
Source: Front Plant Sci. 2026 Mar 16;17:1746220. doi: 10.3389/fpls.2026.1746220 (PMC13033775; doi:10.3389/fpls.2026.1746220)
Supplement: Supplementary Table 1 — Training environment and main hyperparameters of the sprouting image detection model. [file Table1.docx]

## Tables

Table S Training environment and main hyperparameters of the sprouting image detection model

| operating system | Windows10 |
| --- | --- |
| display card (computer) | NVIDIA GeForce RTX 4090 |
| processing unit | Intel（R） Xeon（R） Gold 6248R @ 3.00GHz |
| Epoch | 100 |
| Batch Size | 16 |
| Image Size | 640×640 |
| Momentum | 0.937 |
| Learning Rate | 0.01 |
| Weight Decay | 0.0001 |
| Random Seed | 0 |
